# The association between maternal diabetes and the risk of attention deficit hyperactivity disorder in offspring: an updated systematic review and meta-analysis

**DOI:** 10.1007/s00787-025-02645-5

**Published:** 2025-01-28

**Authors:** Yitayish Damtie, Berihun Assefa Dachew, Getinet Ayano, Abay Woday Tadesse, Kim Betts, Rosa Alati

**Affiliations:** 1 School of Population Health, Faculty of Health Sciences, Curtin University, Bentley, WA Australia; 2 Department of Public Health, College of Medicine and Health Science, Injibara University, Injibara, Ethiopia; 3 enAble Institute, Curtin University, Perth, WA Australia; 4 Institute for Social Sciences Research, The University of Queensland, Brisbane, Australia

**Keywords:** ADHD, Gestational diabetes, Type 1 diabetes, Type 2 diabetes, Systematic review, Meta-analysis

## Abstract

Mixed results have been reported regarding the link between different types of maternal diabetes and attention deficit hyperactivity disorder (ADHD) in offspring. Hence, we conducted a systematic review and meta-analysis to explore these associations. Relevant studies on the subject were retrieved from six major databases, including PubMed, Medline, Embase, Scopus, CINAHL, and PsychINFO. The methodological quality of the included studies was evaluated using the Newcastle-Ottawa Scale, and between-study heterogeneity was assessed using the I2 statistic. Subgroup, sensitivity, and meta-regression analyses were conducted to identify the sources of heterogeneity between studies. In total, seventeen observational studies (five case-control and twelve cohort studies) with 18,063,336 study participants were included in the final analysis. Our random-effects meta-analysis revealed that exposure to any form of maternal diabetes was associated with an increased risk of ADHD in children. Specifically, we observed a heightened risk of ADHD in children exposed to gestational diabetes mellitus, any pre-existing diabetes, pre-existing type 1 diabetes mellitus, and type 2 diabetes mellitus. Our study suggests that children exposed to diabetes during prenatal development are at a higher risk of developing ADHD. These findings underscore the critical importance of early screening and timely interventions for exposed offspring.

**Supplementary Information:**

The online version contains supplementary material available at 10.1007/s00787-025-02645-5.

## Introduction

Attention deficit hyperactivity disorder (ADHD) is a complex neurodevelopmental disorder characterized by a persistent pattern of inattention, impulsivity, and/or hyperactivity, that significantly interferes with familial, academic, and social functioning [1, 2]. It is the most common childhood psychopathology seen in psychiatric clinics [3] and the third leading mental health-related burden of disease in Europe next to anxiety and mood disorders [4]. According to a recent umbrella review, the pooled prevalence of ADHD in children and adolescents was estimated to be 8% [5], with reported pooled estimates ranging from 3.4–13.8% [6–9]. It is more common in boys [5, 10] and often co-occurs with other mental disorders [11–13].

The underlying cause of ADHD remains unclear. While genetic factors are established as the predominant etiological contributors in nearly three-fourth of cases [14], emerging research suggests that prenatal and perinatal factors, including diabetes during pregnancy, may also play a role in its development [15]. Maternal diabetes, characterised by an elevated glucose blood level, represent a prevalent metabolic disorder affecting approximately 16.7% of pregnancies worldwide [16]. Gestational diabetes mellitus (GDM) is the predominant form, responsible for nearly 85% of diabetes cases, while type 1 diabetes (T1DM) and type 2 diabetes (T2DM) account for around 7% and 5% of cases, respectively [17].

Existing studies examining the link between intra-uterine exposure to diabetes and ADHD risk in children have reported inconsistent results. Some studies [18–20] but not all [21–23] have identified an elevated risk. For example, a retrospective cohort study conducted by Perea et al. revealed that children exposed to GDM had a 64% increased risk of developing ADHD compared to unexposed counterparts [18]. Another study conducted by Lee et al. reported a 55% higher risk of ADHD in children exposed to T1DM [24]. Further, a prospective cohort study by Nomura et al. [19] and a recent publication by Lin and colleagues [20] identified a twofold higher ADHD risk in offspring exposed to GDM and any form of maternal diabetes, respectively. In contrast, a population-based retrospective cohort study conducted in Taiwan [21] and another study in the United States [23] found insufficient statistical evidence for the association between T1DM and GDM and ADHD risk in children, respectively, despite adequate adjustment for confounding factors and sufficient sample sizes.

While three previous meta-analyses have examined the link between maternal diabetes and the risk of ADHD in offspring, these studies also reported inconsistent results and have methodological limitations [25–27]. For instance, a meta-analysis conducted by Rowland et al. in 2021 found no association between GDM and ADHD risk in children [27]. This meta-analysis included only five studies and the authors did not perform subgroup, sensitivity, and meta-regression analyses due to the limited number of included studies. The second meta-analysis by Yamamoto et al., 2019 [26] reported a statistically significant association between maternal pre-existing diabetes and ADHD, but this association was based on only two studies. The third meta-analysis by Guo et al., 2020 [25], which included seven studies, identified an elevated risk of ADHD in children exposed to any form of maternal diabetes. However, this review did not distinguish between subcategories of maternal diabetes, specifically T1DM and T2DM. Since the last review [25], seven new articles and three additional studies that were not previously considered, have been identified (Table S1), suggesting the need for updating the existing evidence. It is also important to note that, neither of the previous meta-analyses distinguished between the impact of T1DM and T2DM on the risk of ADHD in offspring.

To address these gaps in the literature, we conducted an updated meta-analysis to examine the relationship between different types of maternal diabetes, including T1DM and T2DM, and the risk of ADHD in offspring. We also performed a cumulative meta-analysis to track the trend of the pooled estimate and its precision over time and explore sources of variation in the results.

## Methods

### Research design and protocol registration

This meta-analysis rigorously followed the guidelines of the Preferred Reporting Items for Systematic Review and Meta-analysis (PRISMA) [28] (Table S1). The study protocol was registered in the International Prospective Register of Systematic Reviews (PROSPERO) with registration number CRD42023461669. Literature searching, article selection, data abstraction, and synthesis were carried out in accordance with the pre-established protocol.

### Data source and search strategy

A systematic literature search was conducted across six major databases—PubMed, Medline/Ovid, Embase/Ovid, Scopus, CINAHL, and PsycINFO/Ovid—to identify relevant studies published from inception up to September 8, 2023. The search strategy included the following search terms: [(maternal diabete* OR diabetic gravida* OR maternal hyperglycaemia OR maternal type 1 diabete* OR maternal type 2 diabete* OR gestational diabete* OR maternal pre-existing diabete* OR maternal metabolic disease* OR immune system disease* OR obstetric complication* OR maternal autoimmune disease* OR prenatal factor* OR perinatal factor*) AND (ADHD OR attention deficit/hyperactivity disorder* OR attention deficit hyperactivity disorder* OR neurodevelopmental disorder* OR developmental disabilit* OR attention deficit OR attention-deficit OR inattent* OR hyperactiv* OR hyperkinetic disorder*) AND (child* OR toddler* OR offspring* OR adolescent* OR adult*)] (Table S2). Two reviewers (YD and AWT) independently screened the titles and abstracts of the identified articles using Rayyan software, and any discrepancies between reviewers were resolved through discussion and consulting a third reviewer. To enhance the comprehensiveness of our literature search, the reference lists of the included articles were manually examined.

### Eligibility criteria

This meta-analysis included all case-control and cohort studies examining the association between maternal diabetes and ADHD in offspring. Studies were eligible if they assessed ADHD using both symptom scales and clinical diagnoses, and maternal diabetes—including GDM, pre-existing T1DM, and T2DM—was identified through self-report, clinical records, and/or diagnostic criteria. Cross-sectional studies were excluded due to their limitations in establishing causal relationships between exposures and outcomes. We also excluded reviews, books, case series, case reports, animal studies, non-English language publications, conference abstracts, and studies that did not report effect estimates—such as odds ratios (OR), relative risks (RR), or hazard ratios (HR) with 95% confidence intervals (CI)—or lacked sufficient data to calculate them.

### Data extraction

For each eligible study, we extracted the following information: the name of the primary author, study country, year of publication, study design, data source, sample size, study period, type of maternal diabetes, method of exposure measurement, outcome ascertainment method, offspring age, adjusted confounders, and effect estimates with their corresponding 95% CI. Any discrepancies encountered during data extraction were resolved through discussion involving a third reviewer.

### Quality assessment

Newcastle-Ottawa Scale for observational studies was utilized to evaluate the study quality [29]. The overall study quality was categorised as low (0–3 points), moderate (4–6 points), and high (7–9 points) based on the number of stars awarded in the selection, comparability, and outcome/exposure ascertainment category. Two independent investigators (Y.D. and A.W.T.) appraised the quality of included studies and any conflicting results were resolved via consensus.

### Data synthesis and analysis

A conventional random-effects meta-analysis was conducted to calculate pooled effect estimates. Additionally, we performed a cumulative meta-analysis to examine how effect estimates evolved over time by sequentially incorporating each newly published article in order of its publication year. For studies reporting multiple exposure data, a combined estimate was used in the main analysis. RR with a 95% CI was used as the measure of association. Non-RR effect estimates (OR and HR) were transformed into RR using the following formulas [30, 31]:

$$\:RR=\frac{OR}{\left(\left(1-r\right)+\left(r*OR\right)\right)}$$

$$\:RR=\frac{1-{e}^{HR\:X\:\text{l}\text{n}(1-r)}}{r}$$

Where, RR is the relative risk, OR is odds ratio, HR is hazard ratio and r is the risk in control or unexposed group. We utilized Higgin I^2^ and Cochran’s Q test to examine the heterogeneity of the included studies [32]. In this meta-analysis, an I^2^ value of zero represents the absence of heterogeneity, while a value of 25%, 50%, and 75% indicate mild, moderate, and high heterogeneity respectively [32]. To identify the potential sources of heterogeneity among studies, we undertook subgroup and meta-regression analysis based on key study level characteristics such as study design, exposure ascertainment method, outcome measurement method, study quality, adjusted confounders, and the type of effect estimate reported. In addition, a leave-one-out sensitivity analysis was conducted to assess the impact of each study on the overall pooled estimate. Finally, potential publication bias was assessed through funnel plot examination and Egger’s test [33], and trim-and-fill analysis was further performed to assess small study effects on the observed estimates [34]. All the statistical analyses were performed using STATA version 17.

## Results

**Fig. 1:**
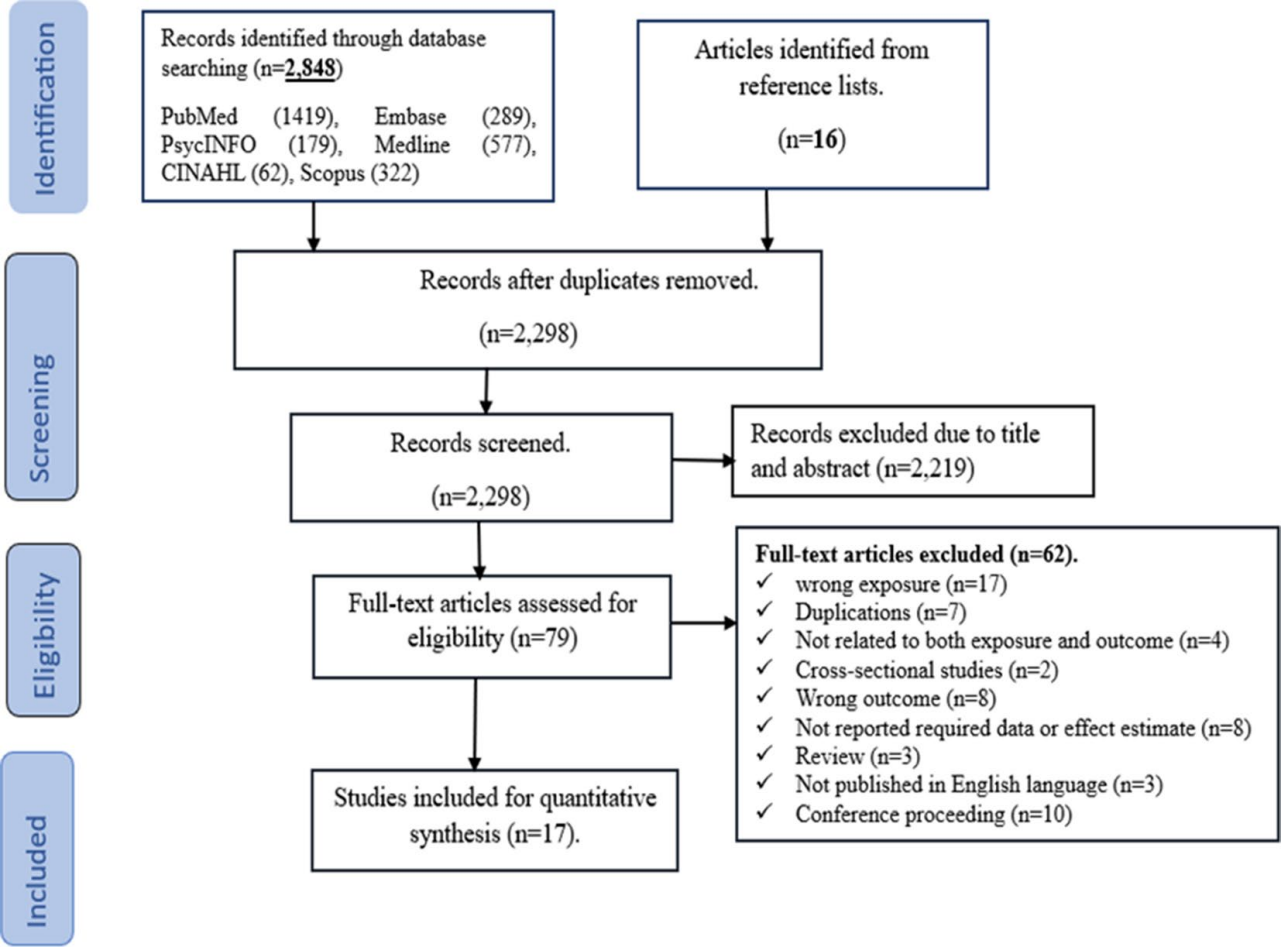
The PRISMA flow diagram

Our initial electronic searches yielded a total of 2,864 articles. Additionally, we retrieved 16 articles through manual searching of reference lists. Of these, 2,785 were excluded due to duplication, while also being irrelevant upon screening their titles and abstracts. The remaining 79 articles underwent full-text assessment for further evaluation. Finally, 17 articles that fulfilled the inclusion criteria were considered in the final analysis (Fig. 1).

### Characteristics of the included studies

Table 1 shows the characteristics of the included studies. In total, 17 articles comprising 26 datasets were included in this systematic review and meta-analysis. Of this, 12 studies were cohort studies, while 5 were case-control studies. The included studies were published between 2012 [35] and 2022 [36] with the sample size ranged from 108 [37] to 2,369,680 [38] study participants. Most of the studies (*n* = 14) made adjustment for at least one potential confounder. Of the included studies, six were conducted in Europe (*n* = 6) [18, 35, 38–42], seven were from Asia (*n* = 7) [21, 24, 37, 43–46], and the remaining four studies were conducted in United States of America (*n* = 4) [36, 47–49]. Fifteen studies utilised diagnostic criteria whereas 2 studies relied on screening and self-report methods to ascertain ADHD in offspring. Among the included studies, eleven studies provided data related to GDM while nine studies provided information about pre-existing diabetes (. Of these, six studies provide data for pre-existing T1DM while four studies for T2DM separately. It is important to note that some studies have simultaneously examined multiple exposures, and therefore, the cumulative count of each exposure may exceed the total number of included studies (Table 1).

**Table 1:** Characteristics of studies included in this systematic review and meta-analysis

Authors: Year	Country of study	Study design	Sample size (total/ ADHD)	Type of exposure evaluated	Exposure measurement	Outcome measurement	Offspring age	Effect estimates (95% CI)
Akaltun et al. [43]	Turkey	Case-control	265/41	Both GDM & PGDM	Based on Carpenter-Coustan’s criteria, file reviews and histories taken from mother	K-SADS-PL	6–12 years	GDM: RR = 2.26 (1.03–4.47) PGDM: RR = 5.23 (2.73–8.35)
Chen K et al. [21]*	China (Taiwan)	Cohort	877,233/not reported	T1DM, T2DM & GDM	ICD-9 codes	ICD-9 codes	Birth to 12 years	T1DM: RR = 1.33 (0.94–1.88) T2DM: RR = 1.29 (1.20–1.39) GDM: RR = 1.07 (1.04–1.10
Chen S et al. [38]*	Sweden	Cohort	2,369,680/102,018	T1DM, T2DM & GDM	ICD-9 & 10 codes	ICD-9 & 10 codes	6–29 years	T1DM: RR = 1.20 (1.12–1.27) T2DM: RR = 1.40 (1.15–1.71) GDM: RR = 1.15 (1.08–1.22)
Cochran et al. [22]*	United States	Cohort	575/87	Any maternal diabetes, type not specified	Maternal interview	MINI-KID	At 15 years	Any DM: RR = 1.51(0.84–2.73)
Halmoy et al. [35]^†^	Norway	Case-control	1,172,396/2,323	Any pre-existing diabetes, type not specified	Medical records, diagnostic standard not specified	ICD-10 codes	Mean age 31.2 years	PGDM: RR = 1.80 (0.80–3.70)
Instanes et al. [51]	Norway	Case-control	2,322,657/47,944	T1DM & T2DM	Medical records, diagnostic standard not specified	Prescribed and dispensed ADHD medications	Not reported	T1DM: RR = 1.49 (1.20–1.87) T2DM: RR = 1.10 (0.70–1.77)
Ji et al. [52]	Sweden	Cohort	1,396,444/21,079	Only T1DM	ICD-8,9 &10 codes	ICD-9 &10 codes	Median age 25 years	T1DM: HR = 1.35 (1.18–155) ^¥^
Lee et al. [24]*	China (Taiwan)	Cohort	708,517/28,209	Only T1DM	ICD-9-CM codes	ICD-9-CM codes	Mean (SD); 6.30 ± 1.78 years	T1DM: RR = 1.53 (1.02–2.30)
Li et al. [53]	Boston	Cohort	2,049/301	Both GDM & PGDM	ICD-9 codes	ICD-9 codes	Median age 5.6 years	GDM: RR = 0.99 (0.52–1.81) PGDM: RR = 1.50 (0.87–2.48)
Lin et al. [53]*	China	Cohort	214/26	Any maternal diabetes, type not specified	Medical records, diagnostic standard not specified	DSM-4, DSM-5	6–10 years	Any DM: RR = 2.28 (1.10–4.24)
Mimouni-Bloch et al. [54]^†^	Israel	Case-control	108/56	Only GDM	Self-report	Medically diagnosed ADHD	6–12 years	GDM: RR = 1.42 (0.62–1.86)
Nomura et al. [19]	New York	Cohort	212/not reported	Only GDM	Face-to-face interview	DSM-IV	6 years	GDM: OR = 2.20 (1.00-4.82) ^¥^
Perea et al. [55]*	Spain	Cohort	3,123/323	Only GDM	Medical records, diagnostic standard not specified	ICD-10 codes	Median age 18.2 years, IQR (14.2–22.3)	GDM: RR = 1.60 (1.30–1.95)
Pohlabeln et al. [42]	Eight European countries^‡^	Cohort	13,355/155	Only GDM	Self-report	Parent-reports	2–11.9 years	GDM: RR = 1.28 (0.59–2.75)
Say et al. [46]	Turkey	Case-control	180/100	Only GDM	Self-report	DSM-IV	3–18 years	GDM: RR = 1.21 (0.27–1.74)
Xiang et al. [50]	United States	Cohort	333,182/17,415	T1DM, T2DM & GDM	ICD-9 codes,	ICD-9 codes	A median age of 4.9 years IQR (2.2, 9.6)	T1DM: RR = 1.54 (1.09–2.18) T2DM: RR = 1.41 (1.27–1.57) GDM: RR = 1.02 (0.96–1.09)
Zhu et al. [47]*	China	Cohort	2,914/186	Only GDM	Medical records, diagnostic standard not specified	C-ASQ	At 3 years	GDM: OR = 0.73 (0.43–1.24) ^¥^

*New publications published after the last systematic review and meta-analysis

^†^Previously published articles not considered in three previous meta-analyses

^‡^Belgium, Cyprus, Estonia, Germany, Hungary, Italy, Spain, and Sweden

^¥^ Transformation of effect estimates was not possible due to insufficient data

C-ASQ = Conners Abbreviated Symptom Questionnaire, DSM = Diagnostic and Statistical Manual of Mental Disorders, GDM = Gestational Diabetes Mellitus, HR = Hazard Ratio, ICD = International Classification of Diseases, IQR = Interquartile Range, K-SADS-PL = Kiddie Schedule for Affective Disorders and Schizophrenia–Present and Lifetime Version, MINI-KID = Mini International Neuropsychiatric Interview for Children and Adolescents, OR = Odds Ratio, PGDM = Pregestational Diabetes Mellitus; RR = Relative Risk, SD = Standard Deviation, T1DM = Type 1 Diabetes Mellitus, T2DM = Type 2 Diabetes Mellitus

### Quality assessment

Most of the included studies (*n* = 12) were rated as high-quality, while the rest 5 studies were deemed to have moderate quality (Table S3).

### Maternal diabetes and risk of ADHD in offspring

**Fig. 2:**
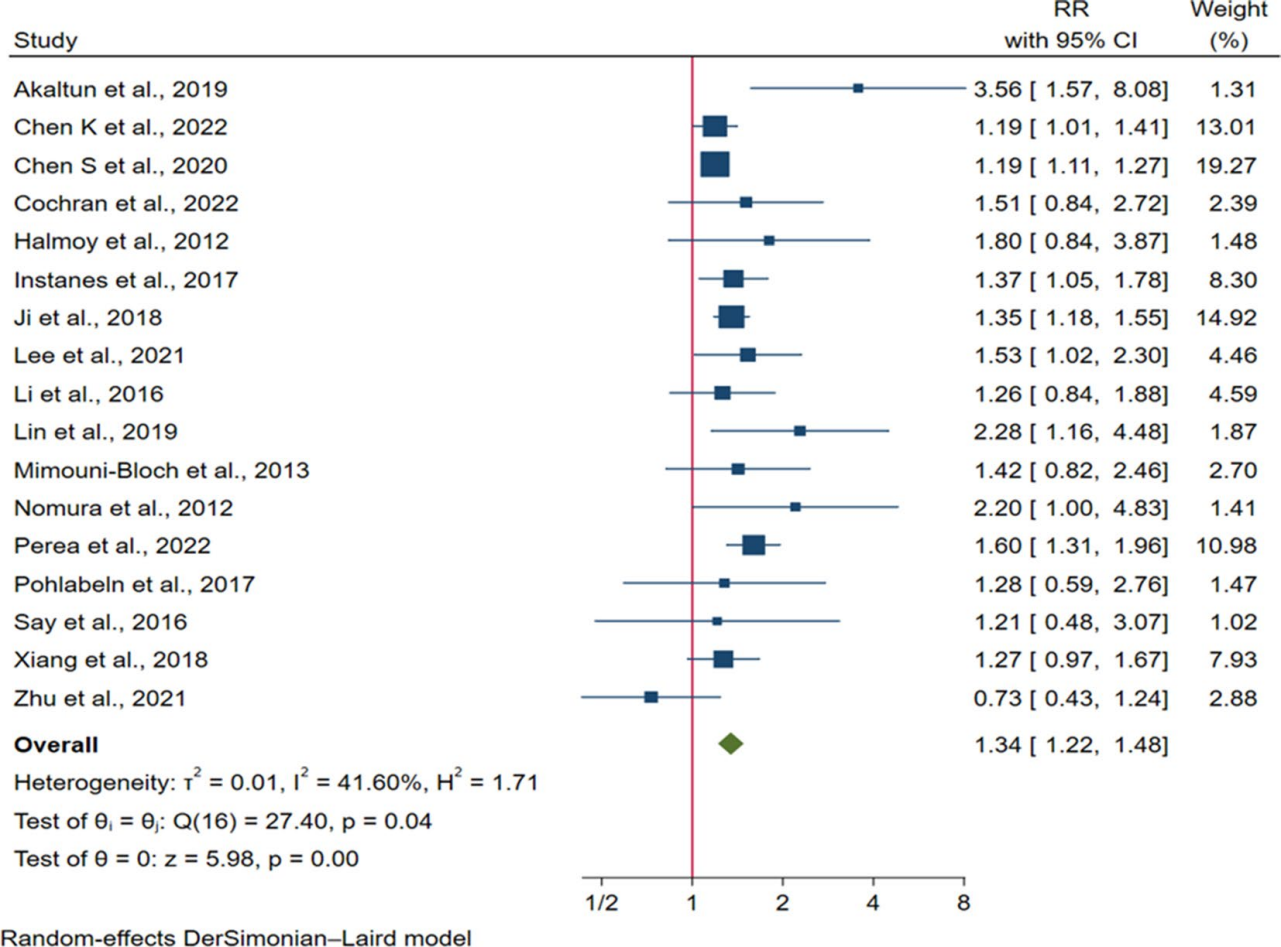
Forest plot showing the risk of ADHD in offspring born to mothers with any form of maternal diabetes

Our random-effects meta-analysis revealed that intra-uterine exposure to any form of maternal diabetes was associated with a 31% increased risk of ADHD in offspring [RR = 1.34: 95% CI: (1.22, 1.48)] (Fig. 2). Our cumulative meta-analysis also yielded consistent results [RR = 1.34: 95% CI: (1.22, 1.48)]. After the publication of a study conducted by Halmoy et al. in 2012 [48], the direction of association remained unchanged despite minimal changes in the pooled RR and their precision (Figure S1).

**Fig. 3:**
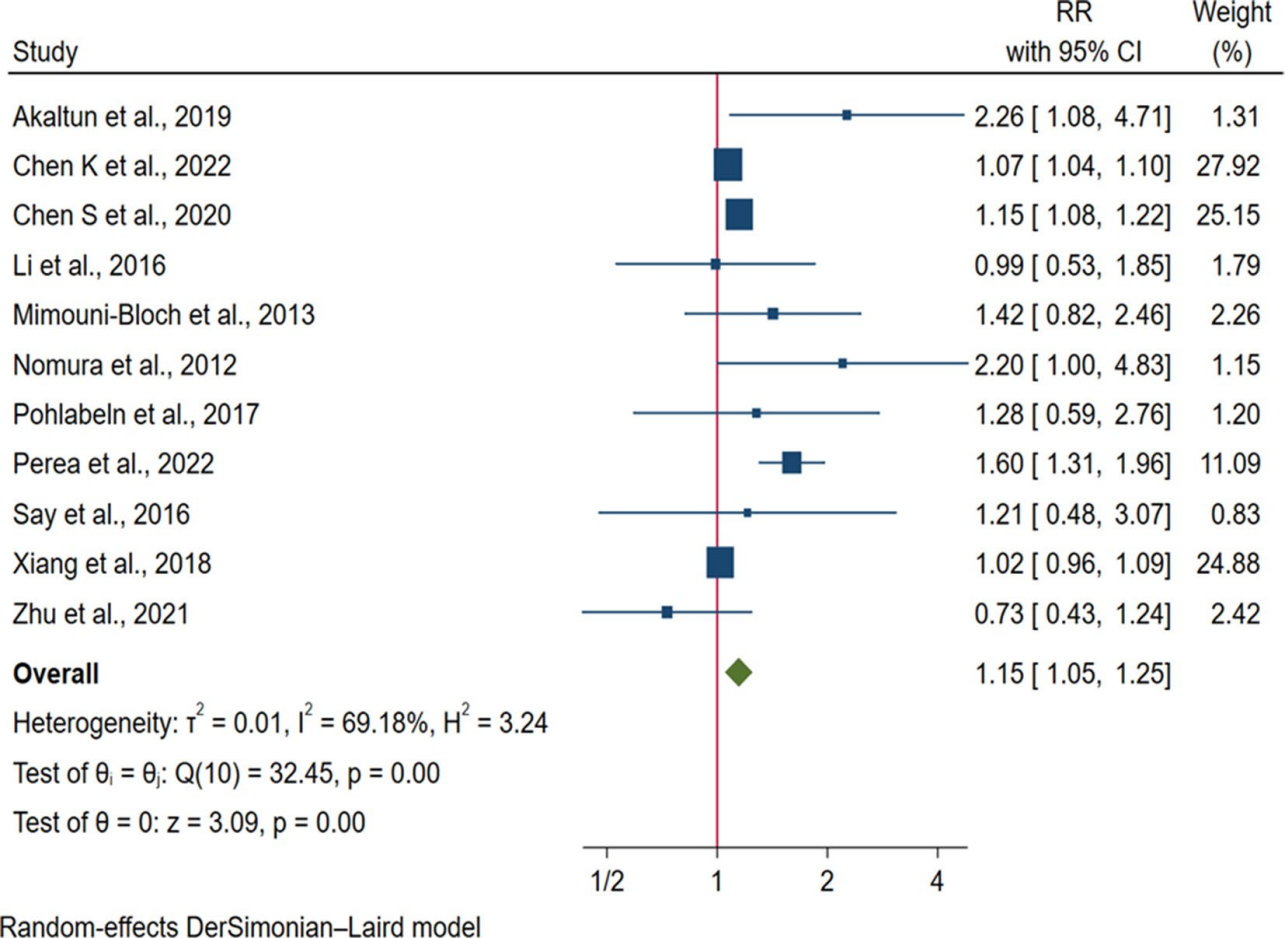
Forest plot showing the pooled effect of gestational diabetes on the risk of ADHD in offspring

**Fig. 4:**
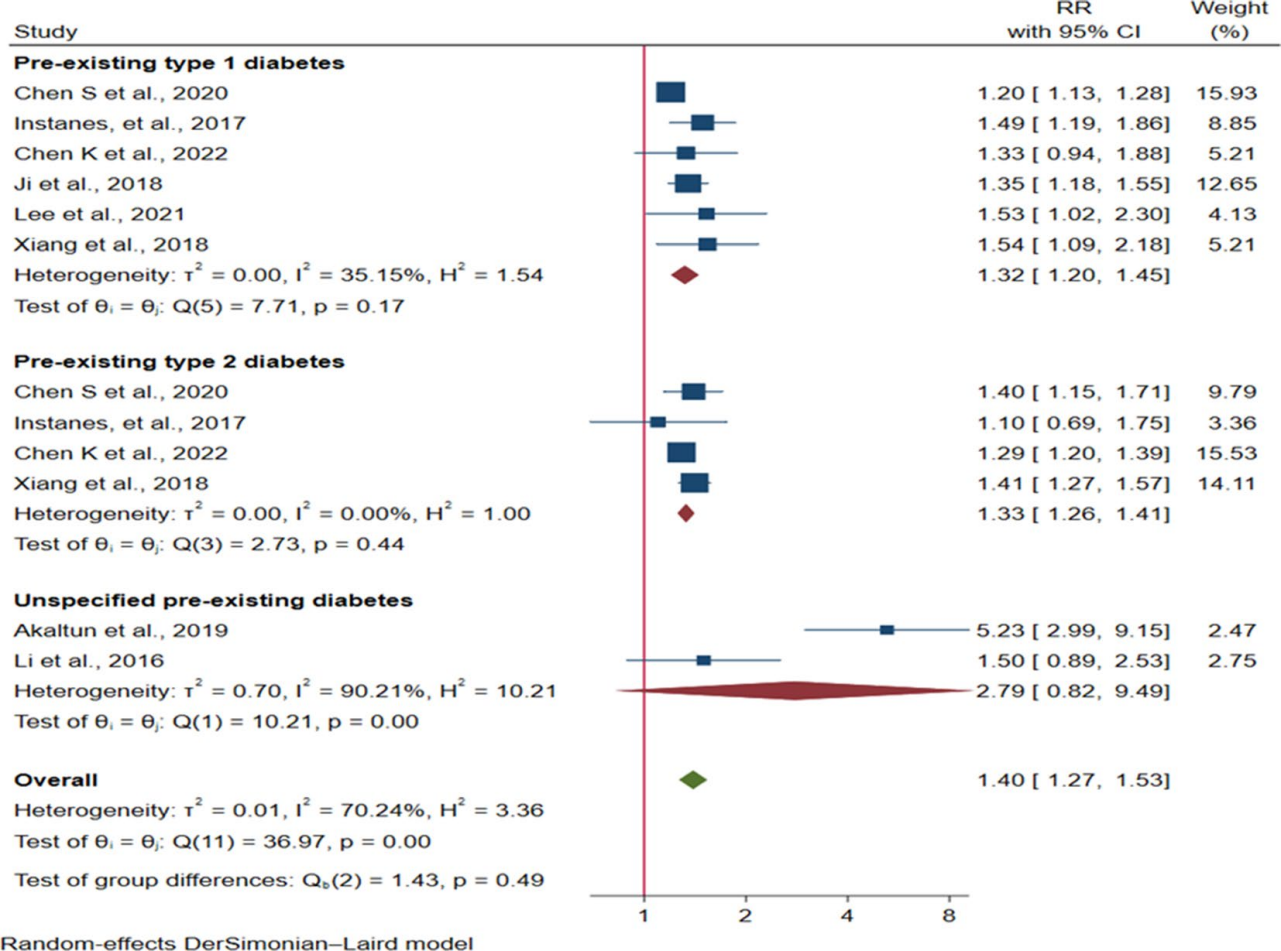
Forest plot showing the effect of any pre-existing diabetes, pre-existing T1DM, and T2DM on ADHD risk in offspring

We also found a 15%, 40%, 32% and 33% increased risk of ADHD in children exposed to GDM [RR = 1.15: 95% CI: (1.05, 1.25)], any pre-existing diabetes [RR = 1.40: 95% CI: (1.27, 1.53)], pre-existing T1DM [RR = 1.32, 95% CI: (1.20, 1.45)] and T2DM [RR = 1.33, 95% CI: (1.26, 1.41)] respectively (Figs. 3 and 4).

### Subgroup and sensitivity analysis

We observed moderate heterogeneity (I² = 41.6%, *P* = 0.04) in studies examining the association between intrauterine exposure to any form of maternal diabetes and the risk of ADHD in offspring. To investigate potential sources of variation, we conducted subgroup analyses based on study design, exposure ascertainment method, outcome ascertainment method, study quality, and the level of confounder adjustment.

In the subgroup analysis, case-control studies demonstrated a higher risk of ADHD (RR = 1.56, 95% CI: 1.17, 2.08) compared to cohort studies (RR = 1.31, 95% CI: 1.19, 1.45). However, the overlapping confidence intervals suggest that this difference may not be statistically significant and should be interpreted with caution. Similarly, studies relying on maternal self-report (RR = 1.66, 95% CI: 1.24, 2.22) and medical records (RR = 1.55, 95% CI: 1.15, 2.09) reported a higher risk of ADHD compared to studies using diagnostic criteria to ascertain maternal diabetes (RR = 1.22, 95% CI: 1.16, 1.29), although the difference was not statistically significant due to overlapping confidence intervals. In contrast, studies using self-report and screening methods to ascertain ADHD in offspring did not show a significant association (RR = 0.90, 95% CI: 0.53, 1.53), whereas studies using diagnostic criteria reported a clearer association (RR = 1.36, 95% CI: 1.24, 1.50) (Table S4).

To address potential confounding effects, we conducted subgroup analyses based on the level of adjustment for maternal age, parental ADHD, maternal socioeconomic status (SES), BMI, and other relevant factors. Studies that did not adjust for maternal age at delivery reported a higher risk of ADHD (RR = 1.90, 95% CI: 1.22, 2.96) compared to studies that accounted for this factor (RR = 1.30, 95% CI: 1.19, 1.42). However, this difference should be interpreted cautiously due to overlapping confidence intervals. The risk of ADHD was comparable between studies that adjusted for parental ADHD (RR = 1.37, 95% CI: 1.22, 1.53) and those that did not (RR = 1.34, 95% CI: 1.17, 1.53), indicating no statistically significant difference between these groups. Similarly, no significant differences were observed between studies adjusting for maternal SES, BMI, and other confounders and those that did not (Table S4).

To assess the robustness of our findings, we conducted a series of sensitivity analyses. Excluding three studies that reported unadjusted estimates yielded a pooled RR of 1.32 (95% CI: 1.20, 1.44) (Figure S2). Similarly, excluding six studies with smaller sample sizes (*n* < 1,000) resulted in consistent findings (RR = 1.29, 95% CI: 1.18, 1.41) (Figure S3) with no evidence of significant publication bias (*P* = 0.7951).

Finally, we performed a leave-one-out sensitivity analysis to determine whether the exclusion of any single study influenced the overall effect of maternal diabetes on the risk of ADHD in offspring. As we iteratively removed each study from the analysis, our result remained consistent with the estimates ranging between 1.30 (95% CI: 1.19, 1.43) and 1.38 (95% CI: 1.24, 1.53), suggesting the robustness of our findings (Figure S4).

### Meta-regression analysis

We conducted univariable and multivariable meta-regression analyses to identify significant sources of heterogeneity among the included studies. In the univariable analysis, we examined several potential covariates, including sample size, study design, exposure ascertainment method, outcome measurement method, adjustment for potential confounders, and study quality. A p-value threshold of < 0.8 was applied to select variables for the multivariable model, consistent with methods used in prior studies [54], and all included variables met the inclusion criteria. In the multivariable model, exposure and outcome ascertainment methods significantly contributed to the observed variation among the included studies, while sample size, study design, study quality, and confounding adjustment did not influence the heterogeneity observed in this study. Covariates in the final model collectively explained 66.4% (R² = 66.4%, *P* < 0.0385) of the variability among the studies (Table S6).

### Publication bias

We observed an asymmetrical distribution of effect estimates upon inspecting the funnel plot (Figure S5). Egger’s test provided weak evidence for publication bias (*P* = 0.0544), suggesting a potential asymmetry in the data. To assess the impact of this bias on the pooled estimates, we conducted a trim-and-fill analysis, and four studies were imputed (Figure S6). Even after accounting for potentially missing studies, the finding remained consistent with the initially observed estimates [RR = 1.26, 95% CI: (1.15, 1.36)], indicating that publication bias did not influence our conclusions.

## Discussion

### Key findings

In this meta-analysis, seventeen observational studies involving 18,063,336 study participants were included. To the best of our knowledge, it is the first study to comprehensively examine the impact of different forms of maternal diabetes on the risk of ADHD in offspring, specifically distinguishing between the two types of pre-existing diabetes. Consistent with the previous two meta-analyses [25, 26], but in contrast to a study conducted by Rowland et al. [27], this study found a 34%, 40% and 14% increased risk of ADHD in children exposed to any form of maternal diabetes, any pre-existing diabetes, and GDM respectively. Furthermore, our study provides novel evidence, revealing a 32% and 33% higher risk of ADHD in children of mothers with T1DM and T2DM, respectively, an aspect not fully addressed in earlier research. The results of our cumulative meta-analysis indicate that the association between maternal diabetes and ADHD risk has been stable since 2012 [35], suggesting that robust statistical evidence supporting this link has been available since that time.

### Possible mechanisms for the association

The mechanism linking maternal diabetes to ADHD in offspring remains unclear, but several potential pathways have been proposed. Genetic studies suggest a familial risk between diabetes and ADHD, indicating overlapping genetic predispositions [55]. Maternal diabetes is associated with a range of inflammatory and metabolic disturbances, including hyperinsulinemia, hyperglycemia, and elevated levels of cholesterol, ketones, amino acids, free radicals, and inflammatory markers such as tumor necrosis factor-alpha, cytokines, and interleukins [56–59]. Such disturbances can disrupt placental and fetal brain function, thereby heightening the likelihood of ADHD and other mental disorders in the offspring. Additionally, children born to diabetic mothers are often found to have lower levels of essential nutrients, including iron and docosahexaenoic acid (DHA) [60, 61]. Both DHA and iron play crucial roles in neurological processes vital to fetal brain development, and deficiencies in these nutrients can impair neurodevelopment, increasing the likelihood of disorders such as ADHD [62, 63]. Additionally, diabetic pregnancies are linked to neonatal complications, including preterm birth, low Apgar scores, small-for-gestational-age status, hypoglycaemia, and increased neonatal intensive care unit admissions [64, 65]. These early life adversities are well-established risk factors for the development of neurodevelopmental disorders, including ADHD, later in childhood [66, 67]. Moreover, maternal diabetes may influence offspring ADHD by altering the epigenetic landscape. Hyperglycemia has been shown to modify DNA methylation and histone acetylation, leading to persistent changes in gene expression that could further elevate the risk of neurodevelopmental disorders [68, 69].

In our meta-analysis, we observed a higher risk of ADHD in offspring of mothers with pre-existing diabetes compared to those with GDM. Several factors may explain this difference. Pre-existing diabetes often presents earlier in life, involves longer exposure to hyperglycaemia, and is typically more severe than GDM, which could result in greater prenatal exposure to the associated metabolic and inflammatory risks [70, 71]. Additionally, pre-existing diabetes is often comorbid with other chronic conditions, such as hypertension, renal disease, and cardiovascular disorders, which could further elevate the risk of ADHD in offspring due to the cumulative effect of multiple health risks [72–74]. The autoimmune nature of pre-existing T1DM further contributes to neurodevelopment through direct immune-mediated mechanisms [75].

### Sources of heterogeneity and interpretation of findings

Our meta-analysis identified moderate heterogeneity (I² = 41.6%) in the association between any form of maternal diabetes and the risk of ADHD in offspring. Meta-regression analyses revealed that the methods used for both exposure and outcome ascertainment significantly contributed to the observed heterogeneity. In contrast, factors such as study design, quality, sample size, and confounder adjustment had a limited impact. Specifically, studies that relied on maternal self-report (*p* = 0.046) and medical records (*p* = 0.039) for ascertaining maternal diabetes reported stronger associations than those using diagnostic criteria. Similarly, studies that used self-report and screening methods to assess ADHD in offspring also showed stronger associations. These differences likely reflect the heightened risk of recall bias, social desirability bias, and potential misclassification biases associated with self-report and medical record-based methods [76, 77], which may lead to an overestimation of the true association.

Despite these findings, a notable portion of the heterogeneity (I² ~ 14%) remains unexplained, suggesting that other unmeasured factors, such as offspring demographic characteristics (e.g., age and sex), may contribute to the variability observed across studies.

While not statistically significant, our subgroup analysis indicated that the risk of ADHD was higher in case-control studies compared to cohort studies. This discrepancy may stem from biases inherent in case-control designs, including selection bias, recall bias, and differential misclassification, which are less common in cohort studies [78]. Additionally, no significant differences in ADHD risk were found based on adjustments for maternal BMI, tobacco use during pregnancy, maternal age at childbirth, or parental ADHD. Although this suggests the robustness of our overall findings, the inconsistent control for confounders across studies means that unmeasured or inadequately controlled factors may still contribute to the heterogeneity observed.

Our sensitivity analysis, excluding studies with crude estimates and smaller sample sizes, yielded consistent results, further supporting the robustness of our conclusions. However, residual heterogeneity and methodological limitations warrant cautious interpretation. Future research should aim to address unmeasured or inadequately controlled factors and rectify the methodological flaws identified in this analysis to enhance our understanding of the association between maternal diabetes and ADHD risk in offspring.

### Strengths and limitations

Our meta-analysis had several strengths. To our knowledge, it is the first to examine the impact of maternal T1DM and T2DM on the risk of ADHD in offspring. We undertook a random effect cumulative meta-analysis to examine the trend of pooled effect estimates as newly published articles were included over time. In addition, detailed subgroup, sensitivity, and meta-regression analyses were performed to explore the source of variability among studies. Furthermore, a standardised quality assessment tool (NOS) was utilized to evaluate the study quality.

This study also had several limitations to consider. Firstly, our inclusion criteria were limited English language publications; important articles published in another language may have been missed. Secondly, we found significant heterogeneity across seventeen included studies (I^2^ = 41.6%); though the robustness of our findings was confirmed through sensitivity, subgroup, sensitivity, and meta-regression analysis. Thirdly, most of the included studies did not account for important confounding variables, leaving open the possibility of chance findings. Furthermore, two of the eighteen studies utilized self-report methods to ascertain ADHD in offspring, which could potentially introduce measurement bias and impact the accuracy of our findings. It is worth noting that the exclusion of such studies from our analysis did not alter our results. Moreover, five out of the 18 studies included in this meta-analysis were of moderate quality. Again, when we excluded them from the analysis, the results remained unchanged. Finally, weak evidence of publication bias was identified through both the funnel plot and Egger’s regression test. However, the trim-and-fill analysis indicated that this bias did not significantly affect our conclusions.

### Implications of the findings

Our findings provide crucial evidence with significant clinical and research implications. The study highlights the need for routine diabetes screening and effective blood glucose management before conception and during pregnancy to mitigate associated risks. Given the differential impact of various forms of diabetes, healthcare providers should not only focus on managing diabetes during pregnancy but also address the unique risks posed by each type. Additionally, considering the heightened risk of ADHD in children born to mothers with diabetes, early screening and timely intervention for neurodevelopmental disorders are vital for minimizing long-term developmental challenges and improve quality of life.

Building upon our findings, future research should investigate the biological mechanisms through which maternal diabetes increases the risk of ADHD in offspring, with particular attention to the risks associated with different subtypes of maternal diabetes. Additionally, high-quality cohort studies that adequately control for important confounders are essential to further validate our findings. Our meta-analysis primarily focused on categorically diagnosed maternal diabetes, and the impact of antidiabetic medication use on offspring ADHD risk was not explored due to limited available data. Therefore, future studies should prioritize this area, as the risk of ADHD may vary based on blood glucose levels and the use of diabetic medications [18].

## Conclusion

Our meta-analysis found that maternal diabetes is associated with an increased risk of ADHD in offspring, with a stronger effect observed in cases of pre-existing diabetes compared to GDM. However, the heterogeneity observed in the results warrants cautious interpretation. Further investigation is needed to elucidate the underlying biological mechanisms linking maternal diabetes to the risk of ADHD in offspring.

## Electronic supplementary material

Below is the link to the electronic supplementary material.

Supplementary Material 1

## Data Availability

No datasets were generated or analysed during the current study.
