# Supplementary material for: The association between maternal diabetes and the risk of attention deficit hyperactivity disorder in offspring: an updated systematic review and meta-analysis
Source: Eur Child Adolesc Psychiatry. 2025 Jan 28;34(8):2417–29. doi: 10.1007/s00787-025-02645-5 (PMC12396981; doi:10.1007/s00787-025-02645-5)
Supplement: Supplementary file 1 — Supplementary Material 1 [file 787_2025_2645_MOESM1_ESM.docx]

**The association between maternal diabetes and the risk of attention deficit hyperactivity disorder in offspring: An updated systematic review and meta-analysis.**

Yitayish Damtie^1,2*^, Berihun Assefa Dachew^1,3^, Getinet Ayano^1^, Abay Woday Tadesse^1^, Kim Betts^1^, Rosa Alati ^1^

1. **List of tables**

**Table S1:** PRISMA-2020 Statement to report the findings of the study.

**Table S2:** Searching strategy for the association between maternal diabetes and risk of attention-deficit/ hyperactivity disorder in offspring, 2023.

**Table S3:** Quality assessment of included studies using the Newcastle-Ottawa Scale.

**Table S4:** Adjusted confounders in studies included in the meta-analysis

**Table S5**: Sensitivity and subgroup analysis for the association between any form of maternal diabetes and risk of ADHD in offspring

**Table S6**: Univariable and multivariable meta-regression analysis for the association between any form of maternal diabetes and risk of ADHD in offspring.

1. **List of figures**

**Figure S1:** Cumulative meta-analysis on the association between maternal diabetes and ADHD in offspring.

**Figure S2: S**ensitivity analysis removing three studies with unadjusted estimates.

**Figure S3: S**ensitivity analysis removing six studies with small sample size (n<1000).

**Figure S4:** leave-one-out sensitivity analysis to assess single study effect on the association between maternal diabetes and the risk of ADHD in offspring.

**Figure S5:** Funnel plot showing publication bias among studies examining any form of maternal diabetes and the risk of ADHD in offspring.

**Figure S6:** Funnel plot showing studies imputed for trim-fill analysis.

1. **List of tables**

**Table S1:** PRISMA-2020 Statement to report the findings of the study.

| **Section and Topic** | **Item #** | **Checklist item** | **Location where item is reported** |
| --- | --- | --- | --- |
| **TITLE** | | |  |
| Title | 1 | Identify the report as a systematic review. | Page 1 |
| **ABSTRACT** | | |  |
| Abstract | 2 | See the PRISMA 2020 for Abstracts checklist. | Page 2 |
| **INTRODUCTION** | | |  |
| Rationale | 3 | Describe the rationale for the review in the context of existing knowledge. | Page 4 |
| Objectives | 4 | Provide an explicit statement of the objective(s) or question(s) the review addresses. | Page 4 |
| **METHODS** | | |  |
| Eligibility criteria | 5 | Specify the inclusion and exclusion criteria for the review and how studies were grouped for the syntheses. | Page 5-6 |
| Information sources | 6 | Specify all databases, registers, websites, organisations, reference lists and other sources searched or consulted to identify studies. Specify the date when each source was last searched or consulted. | Page 5 |
| Search strategy | 7 | Present the full search strategies for all databases, registers and websites, including any filters and limits used. | Page 5 and table S2 |
| Selection process | 8 | Specify the methods used to decide whether a study met the inclusion criteria of the review, including how many reviewers screened each record and each report retrieved, whether they worked independently, and if applicable, details of automation tools used in the process. | Page 5 |
| Data collection process | 9 | Specify the methods used to collect data from reports, including how many reviewers collected data from each report, whether they worked independently, any processes for obtaining or confirming data from study investigators, and if applicable, details of automation tools used in the process. | Page 6 |
| Data items | 10a | List and define all outcomes for which data were sought. Specify whether all results that were compatible with each outcome domain in each study were sought (e.g. for all measures, time points, analyses), and if not, the methods used to decide which results to collect. | Page 6 |
|  | 10b | List and define all other variables for which data were sought (e.g. participant and intervention characteristics, funding sources). Describe any assumptions made about any missing or unclear information. | Page 6 |
| Study risk of bias assessment | 11 | Specify the methods used to assess risk of bias in the included studies, including details of the tool(s) used, how many reviewers assessed each study and whether they worked independently, and if applicable, details of automation tools used in the process. | Page 6 |
| Effect measures | 12 | Specify for each outcome the effect measure(s) (e.g. risk ratio, mean difference) used in the synthesis or presentation of results. | Page 7 |
| Synthesis methods | 13a | Describe the processes used to decide which studies were eligible for each synthesis (e.g. tabulating the study intervention characteristics and comparing against the planned groups for each synthesis (item #5)). | Page 7 |
|  | 13b | Describe any methods required to prepare the data for presentation or synthesis, such as handling of missing summary statistics, or data conversions. | Page 7 |
|  | 13c | Describe any methods used to tabulate or visually display results of individual studies and syntheses. | Page 7 |
|  | 13d | Describe any methods used to synthesize results and provide a rationale for the choice(s). If meta-analysis was performed, describe the model(s), method(s) to identify the presence and extent of statistical heterogeneity, and software package(s) used. | Page 7 |
|  | 13e | Describe any methods used to explore possible causes of heterogeneity among study results (e.g. subgroup analysis, meta-regression). | Page 7 |
|  | 13f | Describe any sensitivity analyses conducted to assess robustness of the synthesized results. | Page 7 |
| Reporting bias assessment | 14 | Describe any methods used to assess risk of bias due to missing results in a synthesis (arising from reporting biases). | Page 7 |
| Certainty assessment | 15 | Describe any methods used to assess certainty (or confidence) in the body of evidence for an outcome. | Page 7 |
| **RESULTS** | | |  |
| Study selection | 16a | Describe the results of the search and selection process, from the number of records identified in the search to the number of studies included in the review, ideally using a flow diagram. | Page 7 & 8 |
|  | 16b | Cite studies that might appear to meet the inclusion criteria, but which were excluded, and explain why they were excluded. | Figure 1 |
| Study characteristics | 17 | Cite each included study and present its characteristics. | Page 8 |
| Risk of bias in studies | 18 | Present assessments of risk of bias for each included study. | Page 12 |
| Results of individual studies | 19 | For all outcomes, present, for each study: (a) summary statistics for each group (where appropriate) and (b) an effect estimate and its precision (e.g. confidence/credible interval), ideally using structured tables or plots. | Page 12, Figure 2-4 |
| Results of syntheses | 20a | For each synthesis, briefly summarise the characteristics and risk of bias among contributing studies. | Page 12-13 |
|  | 20b | Present results of all statistical syntheses conducted. If meta-analysis was done, present for each the summary estimate and its precision (e.g. confidence/credible interval) and measures of statistical heterogeneity. If comparing groups, describe the direction of the effect. | Page 12, Figure 2-4 |
|  | 20c | Present results of all investigations of possible causes of heterogeneity among study results. | Page 12-13, Table S5 & S6 |
|  | 20d | Present results of all sensitivity analyses conducted to assess the robustness of the synthesized results. | Page 12-13, Table S6 |
| Reporting biases | 21 | Present assessments of risk of bias due to missing results (arising from reporting biases) for each synthesis assessed. | -- |
| Certainty of evidence | 22 | Present assessments of certainty (or confidence) in the body of evidence for each outcome assessed. | -- |
| **DISCUSSION** | | |  |
| Discussion | 23a | Provide a general interpretation of the results in the context of other evidence. | Page 15-17 |
|  | 23b | Discuss any limitations of the evidence included in the review. | Page 18 |
|  | 23c | Discuss any limitations of the review processes used. | Page 18 |
|  | 23d | Discuss implications of the results for practice, policy, and future research. | Page 18 & 19 |
| **OTHER INFORMATION** | | |  |
| Registration and protocol | 24a | Provide registration information for the review, including register name and registration number, or state that the review was not registered. | Page 5 |
|  | 24b | Indicate where the review protocol can be accessed, or state that a protocol was not prepared. | Page 5 |
|  | 24c | Describe and explain any amendments to information provided at registration or in the protocol. | Page 5 |
| Support | 25 | Describe sources of financial or non-financial support for the review, and the role of the funders or sponsors in the review. | Page 19 |
| Competing interests | 26 | Declare any competing interests of review authors. | Page 19 |
| Availability of data, code and other materials | 27 | Report which of the following are publicly available and where they can be found: template data collection forms; data extracted from included studies; data used for all analyses; analytic code; any other materials used in the review. | Page 19 |

**Table S2:** Searching strategy for the association between maternal diabetes and risk of attention-deficit/ hyperactivity disorder in offspring, 2023.

| Database | s.no | Headings, key terms with proximity operators | Result | Date |
| --- | --- | --- | --- | --- |
| PubMed | #1 | "Diabetes Mellitus"[MeSH Terms] OR "diabetes, gestational"[MeSH Terms] OR "diabetes mellitus, type 1"[MeSH Terms] OR "diabetes mellitus, type 2"[MeSH Terms] | 510,042 | 08/09/2023 |
|  | #2 | "maternal diabete*"[All Fields] OR "diabetic gravida*"[All Fields] OR "maternal hyperglycaemia"[All Fields] OR "maternal pre-existing diabete*"[All Fields] OR "maternal type 1 diabete*"[All Fields] OR "maternal type 2 diabete*"[All Fields] OR "gestational diabete*"[All Fields] OR "maternal metabolic disease*"[All Fields] OR "maternal autoimmune disease*"[All Fields] OR "immune system disease*"[All Fields] OR "obstetric complication*"[All Fields] OR "Prenatal factor*"[All Fields] OR "perinatal factor*"[All Fields] | 45,859 | 08/09/2023 |
|  | #3 | #1 or #2 | 538,278 | 08/09/2023 |
|  | #4 | "child*"[All Fields] OR "toddler*"[All Fields] OR "adolescent*"[All Fields] OR "offspring*"[All Fields] OR "adult*"[All Fields] | 9,363,012 | 08/09/2023 |
|  | #5 | "Attention Deficit Disorder with Hyperactivity"[MeSH Terms] OR "Neurodevelopmental Disorders"[MeSH Terms] | 210,287 | 08/09/2023 |
|  | #6 | "ADHD"[All Fields] OR "attention deficit/hyperactivity disorder*"[All Fields] OR "neurodevelopmental disorder*"[All Fields] OR "attention deficit hyperactivity disorder*"[All Fields] OR "developmental disabilit*"[All Fields] OR "attention-deficit"[All Fields] OR "attention deficit"[All Fields] OR "inattent*"[All Fields] OR "hyperactiv*"[All Fields] OR "hyperkinetic disorder*"[All Fields] | 140,300 | 08/09/2023 |
|  | #7 | #5 or #6 | 278,518 | 08/09/2023 |
|  | #8 | #3 and #4 and #7 | **1,419** | 08/09/2023 |
| Embase  (Ovid platform) | #1 | exp maternal diabetes mellitus/ or exp pregnancy diabetes mellitus/ | 47052 | 08/09/2023 |
|  | #2 | ("maternal diabete*" or "diabetic gravida*" or "maternal hyperglycaemia" or "maternal pre-existing diabete*" or "maternal type 1 diabete*" or "maternal type 2 diabete*" or "gestational diabete*" or "maternal Metabolic disease*" or "maternal autoimmune disease*" or "Immune System Disease*," or "obstetric complication*" or "Prenatal factor*" or "perinatal factor*").tw,ab. | 45317 | 08/09/2023 |
|  | #3 | #1 or #2 | 61218 | 08/09/2023 |
|  | #4 | exp attention deficit disorder/ or exp attention deficit hyperactivity disorder/ | 9906 | 08/09/2023 |
|  | #5 | ("attention deficit hyperactivity disorder*" adj5 (child* or toddler* or offspring* or adolescent* or adult*)).tw,ab. | 13468 | 08/09/2023 |
|  | #6 | ("ADHD" or "attention deficit/hyperactivity disorder*" or "neurodevelopmental disorder* or attention deficit hyperactivity disorder*" or "developmental Disabilit*" or "attention deficit" or "attention-deficit" or "Inattent*" or "hyperactiv*" or "hyperkinetic disorder*").tw,ab. | 123545 | 08/09/2023 |
|  | #7 | #4 or #5 or #6 | 126575 | 08/09/2023 |
|  | #8 | #3 and #7 | **289** | 08/09/2023 |
| Medline  (Ovid platform) | #1 | exp Diabetes Mellitus, Type 1/ or exp Diabetes, Gestational/ or exp Diabetes Mellitus, Type 2/ | 257,654 | 08/09/2023 |
|  | #2 | ("maternal diabete*" or "diabetic gravida*" or "maternal hyperglycaemia" or "maternal pre-existing diabete*" or "maternal type 1 diabete*" or "maternal type 2 diabete*" or "gestational diabete*" or "maternal metabolic disease*" or "maternal autoimmune disease*" or "immune system disease*," or "obstetric complication*" or "Prenatal factor*" or "perinatal factor*").tw,ab. | 29668 | 08/09/2023 |
|  | #3 | #1 or #2 | 272784 | 08/09/2023 |
|  | #4 | exp attention deficit disorder with Hyperactivity/ | 34869 | 08/09/2023 |
|  | #5 | ("attention deficit hyperactivity disorder*" adj5 (child* or toddler* or offspring* or adolescent* or adult*)).tw,ab | 10756 | 08/09/2023 |
|  | #6 | ("ADHD" or "attention deficit/hyperactivity disorder*" or "neurodevelopmental disorder* or attention deficit hyperactivity disorder*" or "developmental Disabilit*" or "attention deficit" or "attention-deficit" or "Inattent*" or "hyperactiv*" or "hyperkinetic disorder*").tw,ab | 89484 | 08/09/2023 |
|  | #7 | #4 or #5 or #6 | 94941 | 08/09/2023 |
|  | #8 | #3 and #7 | **577** | 08/09/2023 |
| PsychINFO (Ovid) | #1 | exp Type 2 Diabetes/ or exp Diabetes/ or exp Gestational Diabetes/ | 20941 | 08/09/2023 |
|  | #2 | ("maternal diabete*" or "diabetic gravida*" or "maternal hyperglycaemia" or "maternal pre-existing diabete*" or "maternal type 1 diabete*" or "maternal type 2 diabete*" or "gestational diabete*" or "maternal metabolic disease*" or "maternal autoimmune disease*" or "Immune System Disease*," or "obstetric complication*" or "Prenatal factor*" or "perinatal factor*").tw,ab. | 2058 | 08/09/2023 |
|  | #3 | #1 or #2 | 22501 | 08/09/2023 |
|  | #4 | exp attention deficit disorder with Hyperactivity/ or exp Hyperactivity/ | 34235 | 08/09/2023 |
|  | #5 | ("attention deficit hyperactivity disorder*" adj5 (child* or toddler* or offspring* or adolescent* or adult*)).tw,ab. | 10958 | 08/09/2023 |
|  | #6 | ("ADHD" or "attention deficit/hyperactivity disorder*" or "neurodevelopmental disorder* or attention deficit hyperactivity disorder*" or "developmental Disabilit*" or "attention deficit" or "attention-deficit" or "Inattent*" or "hyperactiv*" or "hyperkinetic disorder*").tw,ab | 70625 | 08/09/2023 |
|  | #7 | #4 or #5 or #6 | 71696 | 08/09/2023 |
|  | #8 | #3 and #7 | **179** | 08/09/2023 |
| Scopus | #1 | TITLE-ABS-KEY ("maternal diabete*" OR "diabetic gravida*" OR "maternal hyperglycaemia" OR "maternal pre-existing diabete*" OR "maternal type 1 diabete*" OR "maternal type 2 diabete*" OR "gestational diabete*" OR "maternal Metabolic disease*" OR "maternal autoimmune disease*" OR "Immune System Disease*," OR "obstetric complication*" OR "Prenatal factor*" OR "perinatal factor*") | 45,627 | 08/09/2023 |
|  | #2 | TITLE-ABS-KEY (child* OR toddler OR offspring* OR adolescent* OR adult*) | 12,562,607 | 08/09/2023 |
|  | #3 | TITLE-ABS-KEY (“ADHD" OR "attention deficit/hyperactivity disorder*" OR "neurodevelopmental disorder* or attention deficit hyperactivity disorder*" OR "developmental Disabilit*" OR "attention deficit" OR "attention-deficit" OR "Inattent*" OR "hyperactiv*" OR "hyperkinetic disorder*”) | 168,152 | 08/09/2023 |
|  | #4 | #1 and #2 and #3 | **322** | 08/09/2023 |
| CINAHL (EBSCO | #1 | MM ("Maternal diabetes") OR MM ("diabetes mellitus, gestational") | 6,756 | 08/09/2023 |
|  | #2 | TI ("maternal diabete*" or "diabetic gravida*" or "maternal hyperglycaemia" or "maternal pre-existing diabete*" or "maternal type 1 diabete*" or "maternal type 2 diabete*" or "gestational diabete*" or "maternal Metabolic disease*" or "maternal autoimmune disease*" or "Immune System Disease*," or "obstetric complication*" or "Prenatal factor*" or "perinatal factor*")  OR  AB ("maternal diabete*" or "diabetic gravida*" or "maternal hyperglycaemia" or "maternal pre-existing diabete*" or "maternal type 1 diabete*" or "maternal type 2 diabete*" or "gestational diabete*" or "maternal Metabolic disease*" or "maternal autoimmune disease*" or "Immune System Disease*," or "obstetric complication*" or "Prenatal factor*" or "perinatal factor*") | 12,685 | 08/09/2023 |
|  | #3 | #1 or #2 | 13,620 | 08/09/2023 |
|  | #4 | TI (child* or toddler* or offspring* or adolescent* or adult*) OR  AB (child* or toddler* or offspring* or adolescent* or adult*) | 1,094,182 | 08/09/2023 |
|  | #5 | MW (attention deficit hyperactivity disorder) OR MW (attention deficit disorder with hyperactivity or adhd) | 19,253 | 08/09/2023 |
|  | #6 | TI (("ADHD" or "attention deficit/hyperactivity disorder*" or "neurodevelopmental disorder* or attention deficit hyperactivity disorder*" or "developmental Disabilit*" or "attention deficit" or "attention-deficit" or "Inattent*" or "hyperactiv*" or "hyperkinetic disorder*")) OR  AB (("ADHD" or "attention deficit/hyperactivity disorder*" or "neurodevelopmental disorder* or attention deficit hyperactivity disorder*" or "developmental Disabilit*" or "attention deficit" or "attention-deficit" or "Inattent*" or "hyperactiv*" or "hyperkinetic disorder*")) | 30,172 | 08/09/2023 |
|  | #7 | # 5 or #6 | 35,323 | 08/09/2023 |
|  | 8 | # 3and #4 and #7 | **62** |  |

**Table S3:** Quality assessment of included studies using the Newcastle-Ottawa Scale.

1. **Cohort studies (n=12)**

| **Author, year** | Representativeness of exposed group (⋆) | Selection of non-exposed group (⋆) | Ascertainment of exposure (⋆) | Demonstrate that the outcome of interest was not present at the start of the study (⋆) | Comparability (⋆⋆) | Assessment of outcome (⋆) | Follow-up long enough for outcomes to occur (⋆) | Adequacy of follow-up of cohorts (⋆) | Total (9⋆) | Overall quality |  |
| --- | --- | --- | --- | --- | --- | --- | --- | --- | --- | --- | --- |
|  |  |  |  |  |  |  |  |  |  |  |  |
| Chen K et al., 2022 | * | * | * |  | ** | * | * | * | 8 | High |  |
| Chen S et al., 2020 | * | * | * |  | ** | * | * | * | 8 | High |  |
| Cochran et al., 2022 | * | * |  | * | ** | * | * | * | 8 | High |  |
| Ji et al., 2018 | * | * | * |  | ** | * | * | * | 8 | High |  |
| Lee et al., 2021 | * | * | * |  | ** | * |  | * | 7 | High |  |
| Li et al.,2016 | * | * | * |  | ** | * |  | * | 7 | High |  |
| Lin et al., 2019 | * | * | * |  | * | * | * | * | 7 | High |  |
| Nomura et al., 2012 | * |  |  | * | ** | * |  | * | 6 | Moderate |  |
| Perea et al., 2022 | * | * | * |  | * | * | * | * | 7 | High |  |
| Pohlabeln et al., 2017 | * | * |  | * | ** |  | * | * | 7 | High |  |
| Xiang et al., 2018 | * | * | * |  | ** | * | * | * | 8 | High |  |
| Zhu et al., 2021 | * | * | * | * | ** | * |  | * | 8 | High |  |

1. **Case-control studies (n=5)**

| **Author, year** | Representativeness of cases (⋆) | Selection of controls (⋆) | Definitions of cases (⋆) | Definitions of controls (⋆) | Comparability (⋆⋆) | Assessment of exposure (⋆) | Same method to ascertain cases and controls (⋆) | Non-response rate (⋆) | Total (9⋆) | Overall quality |  |
| --- | --- | --- | --- | --- | --- | --- | --- | --- | --- | --- | --- |
|  |  |  |  |  |  |  |  |  |  |  |  |
| Akaltun et al., 2019 | * |  | * | * |  | * | * |  | 5 | Moderate |  |
| Halmoy et al., 2012 | * |  | * | * | ** | * | * |  | 7 | High |  |
| Instanes, et al., 2017 | * |  | * |  | ** | * | * |  | 6 | Moderate |  |
| Mimouni-Bloch et al.,2013 | * |  | * | * |  |  | * | * | 5 | Moderate |  |
| Say et al., 2016 | * |  | * | * |  |  | * | * | 5 | Moderate |  |

**Table S4:** Adjusted confounders in studies included in the meta-analysis.

| **Authors: Year** | Adjusted confounders |
| --- | --- |
| Akaltun et al, 2019[41] | Not adjusted |
| Chen K et al, 2022[21] | Maternal age, paternal age, year of birth, child sex, income, urbanization level, hypertensive disorder, and preterm delivery |
| Chen S et al, 2021[36] | Birth year, children’s sex, maternal age, parity, the highest parental educational level, parental income quintile at birth, children’s birthplace in Sweden, parental immigration status, parental history of inpatient psychiatric care, smoking during pregnancy, polycystic ovarian syndrome, and pre-gestational BMI |
| Cochran et al, 2022[22] | Maternal age category, maternal educational status, use of food stamps, use of public insurance, and marital status, as well as family history of ADHD |
| Halmoy et al, 2012[33] | Year of birth, parity, age of mother at birth, educational level of mother, and marital status |
| Instanes et al, 2017[49] | Year of birth, mother’s age at birth, mother’s educational level, mother’s marital status, maternal and paternal use of ADHD medication, birth weight, gestational age |
| Ji et al, 2018[50] | Year at birth, sex, parental history of ADHD and other psychiatric disorders, parental education, income, small for gestational age, maternal smoking and urinary tract infection during pregnancy, low Apgar score, and hypoglycaemia. |
| Lee et al, 2021[24] | Income level, residence, maternal ages, maternal mental disorders, and sex of child |
| Li et al, 2016[51] | Child year of birth, child gender, maternal age, parity, smoking during pregnancy, and preterm birth |
| Lin et al, 2019[51] | Gestational age, birth body weight, days of hospitalization |
| Mimouni-Bloch et al, 2013[52] | Not adjusted |
| Nomura et, 2012[19] | The ages of the mother, child, sex, race/ethnicity, and LBW, self-reports of maternal and paternal ADHD symptoms, maternal alcohol use, and smoking during pregnancy |
| Perea et al, 2022[54] | Maternal age, weeks of gestation, caesarean section, Apgar<6 at 5 minutes after birth, and birth weight |
| Pohlabeln et al, 2017[40] | Child sex, maternal age, SES, and country as random effect, Alcohol during pregnancy, Smoking during pregnancy, Gestational hypertension, Proteinuria, Glycosuria, C-section, Preterm birth, low birth weight, Breastfeeding, Respiratory problems, Infections |
| Say et al, 2016[44] | Not adjusted |
| Xiang et al, 2018 [50] | Random sibling effect, birth year, maternal age at delivery, parity, education, household income, maternal race/ethnicity, history of comorbidity, history of maternal ADHD, and sex of the child |
| Zhu et al, 2021[45] | Maternal pre-pregnancy BMI, hypertensive disorders of pregnancy, maternal age, place of residence, educational level, average monthly income, parity, smoking, foetal sex, birth weight, delivery mode, and gestational age at birth. |

**Table S5:** Sensitivity and subgroup analysis for the association between any form of maternal diabetes (overall) and risk of ADHD in offspring

| Variables | Category | Number of studies | RR (95% CI) | I^2^ | P-value from Q-test |
| --- | --- | --- | --- | --- | --- |
| Study design | Case-control | 5 | 1.56 (1.17, 2.08) | 22.9% | 0.27 |
|  | Cohort | 12 | 1.31 (1.19, 1.45) | 44.2% | 0.05 |
| Exposure measurement | Diagnostic criteria | 6 | 1.22 (1.16, 1.29) | 0.00% | 0.55 |
|  | Maternal self-report | 6 | 1.66 (1.24, 2.22) | 2.3% | 0.40 |
|  | Medical records | 6 | 1.55 (1.15, 2.09) | 64.1 % | 0.02 |
| Outcome measurement | Diagnostic criteria | 15 | 1.36 (1.24, 1.50) | 39.8% | 0.06 |
|  | Self-report &screening method | 2 | 0.90 (0.53, 1.53) | 28% | 0.24 |
| Confounding adjustment | Adjusted | 14 | 1.32 (1.20, 1.44) | 38.1% | 0.07 |
|  | Crude/unadjusted | 3 | 1.80 (0.97, 3.34) | 50.5% | 0.13 |
| Adjusted for maternal age | Adjusted | 13 | 1.30 (1.19, 1.42) | 33.3% | 0.12 |
|  | Not adjusted | 4 | 1.90 (1.22, 2.96) | 33.4% | 0.21 |
| Adjusted for maternal SES | Adjusted | 10 | 1.24 (1.16, 1.32) | 8.6% | 0.36 |
|  | Not adjusted | 7 | 1.63 (1.33, 2.00) | 17.2% | 0.30 |
| Adjusted for parental ADHD | Adjusted | 5 | 1.37 (1.22, 1.53) | 0.00% | 0.71 |
|  | Not adjusted | 12 | 1.34 (1.17, 1.53) | 51.8% | 0.02 |
| Adjusted for alcohol use | Adjusted | 2 | 1.67 (0.96, 2.89) | 0.00% | 0.33 |
|  | Not adjusted | 15 | 1.33 (1.21, 1.47) | 45% | 0.03 |
| Adjusted for pre-pregnancy BMI | Adjusted | 2 | 1.00 (0.64, 1.58) | 68.9% | 0.07 |
|  | Not adjusted | 15 | 1.39 (1.27, 1.51) | 8.4% | 0.36 |
| Adjusted for maternal smoking | Adjusted | 6 | 1.24 (1.09, 1.41) | 40.9% | 0.13 |
|  | Not adjusted | 11 | 1.43 (1.26, 1.63) | 26.3% | 0.19 |
| Quality score | Moderate | 5 | 1.63 (1.19, 2.23) | 32% | 0.21 |
|  | High | 12 | 1.30 (1.18, 1.44) | 40.8% | 0.07 |
| Key: BMI= Body Mass Index, SES= Scio-economic Status, OR=Odds Ratio, HR=Hazard Ratio, RR=Relative Risk | | | | |  |

**Table S6**: Univariable and multivariable meta-regression analysis for the association between any form of maternal diabetes and risk of ADHD in offspring.

| Categories | Crude Exp (b) (95% CI) | p-value | Adjusted Exp (b) (95% CI) | p-value |
| --- | --- | --- | --- | --- |
| Sample size | 0.99 (0.98, 1.00) | 0.193 | 1 (0.99, 1.00) | 0.409 |
| Study design |  |  |  |  |
| Cohort | Reference |  | Reference |  |
| Case-control | 1.16 (0.90, 1.52) | 0.270 | 1.19 (0.94, 1.89) | 0.252 |
| Exposure ascertainment |  |  |  |  |
| ICD codes | Reference |  | Reference |  |
| Medical records | 1.15 (1.05, 1.51) | 0.031 | 1.53 (1.09, 1.60) | 0.039 |
| Self-reports | 1.33 (1.07, 1.91) | 0.027 | 1.47 (1.04, 2.41) | 0.046 |
| Outcome measurement |  |  |  |  |
| Diagnostic criteria | Reference |  | Reference |  |
| Self-report and screening method | 1.65 (1.10, 1.89) | 0.021 | 1.51 (1.06, 1.83) | 0.045 |
| Adjusted for at least one confounder |  |  |  |  |
| Adjusted | Reference |  | Reference |  |
| Not adjusted | 1.32 (0.95, 2.05) | 0.756 | 1.65 (0.97, 1.91) | 0.873 |
| Quality score |  |  |  |  |
| High | Reference |  | Reference |  |
| Moderate | 1.19 (0.91, 1.55) | 0.208 | 0.95 (0.51, 1.77) | 0.883 |
| Test of the model: R^2^ = 66.4%; P-value < 0.0385 | | | | |
| Key: ICD; International Classification of Diseases | | | | |

**2. List of figures**


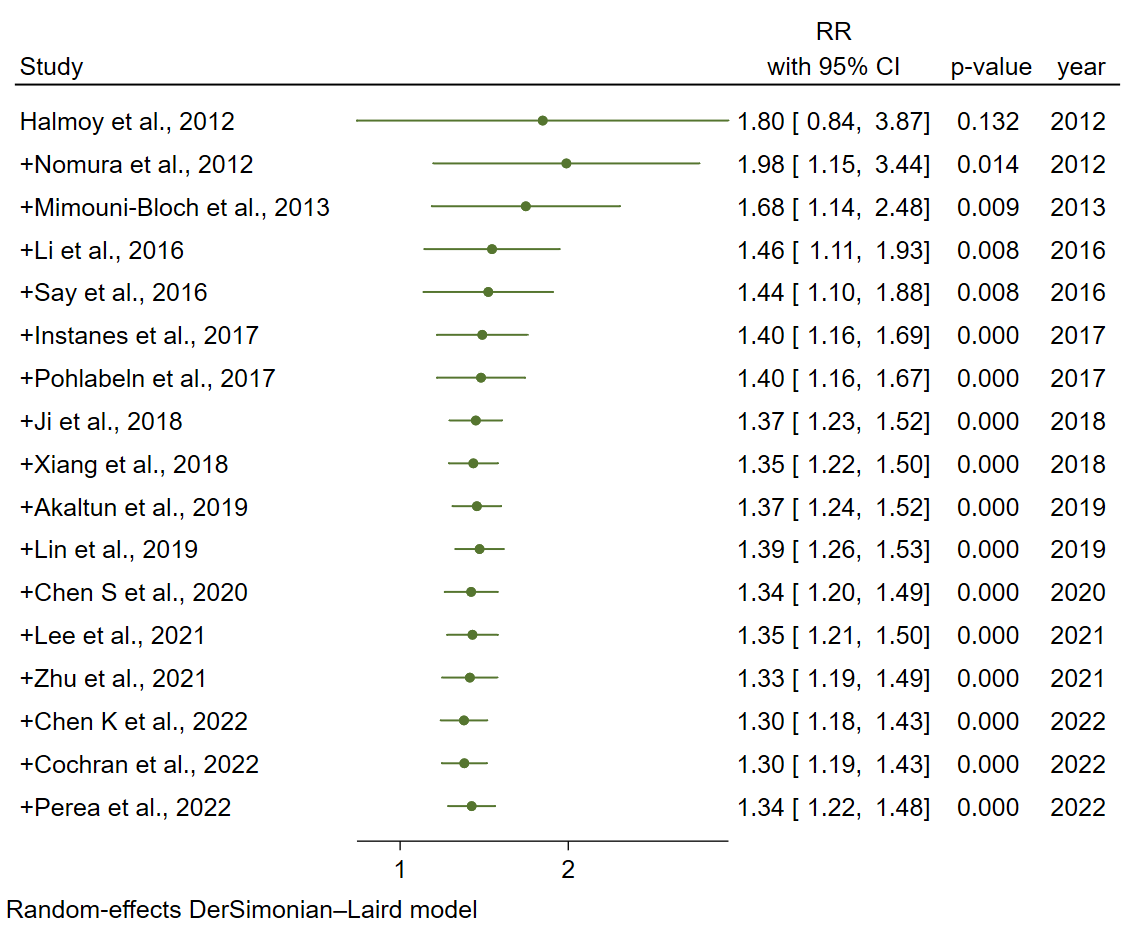


**Figure S1:** Cumulative meta-analysis on the association between maternal diabetes and ADHD in offspring. The '+' symbol indicates the sequential addition of the study results to those previously published.


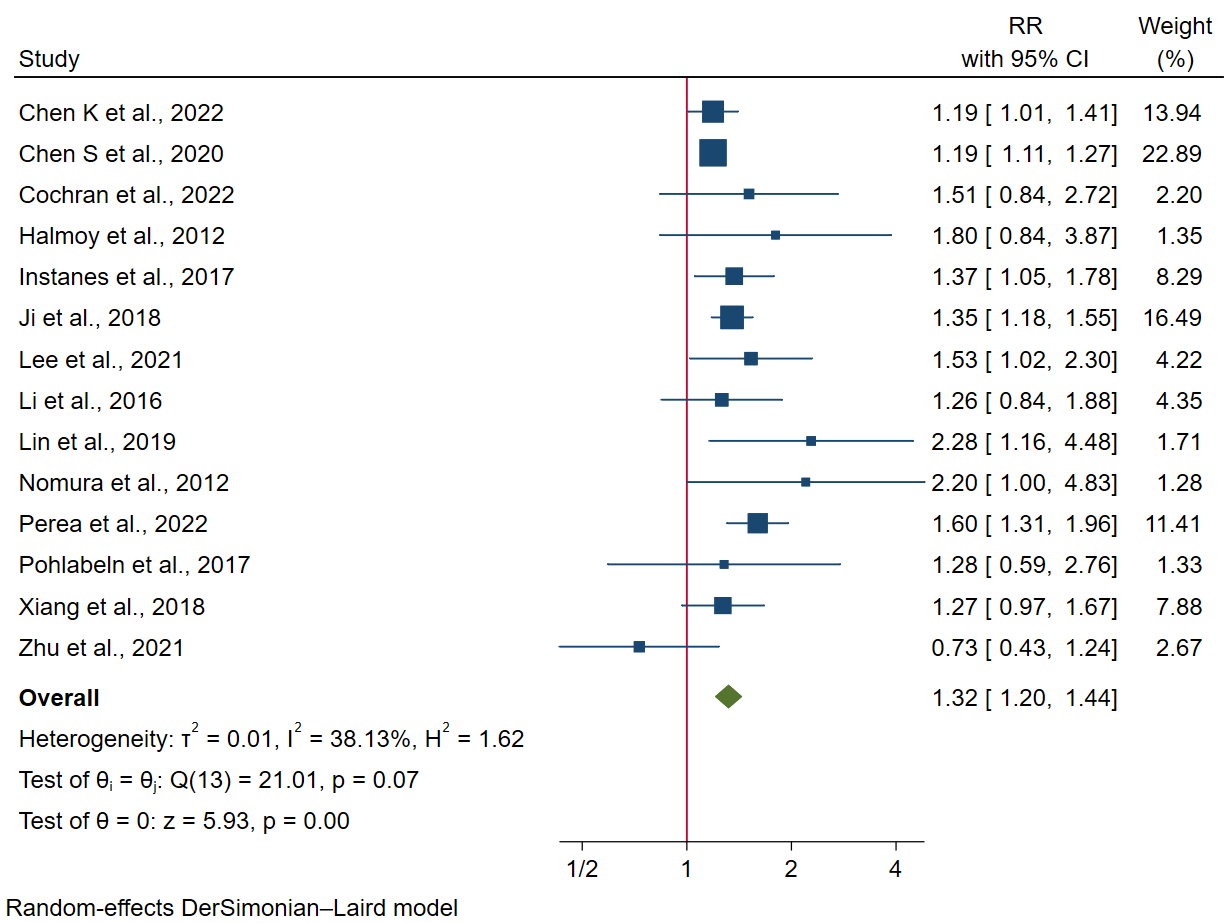


**Figure S2: S**ensitivity analysis removing three studies with unadjusted estimates.


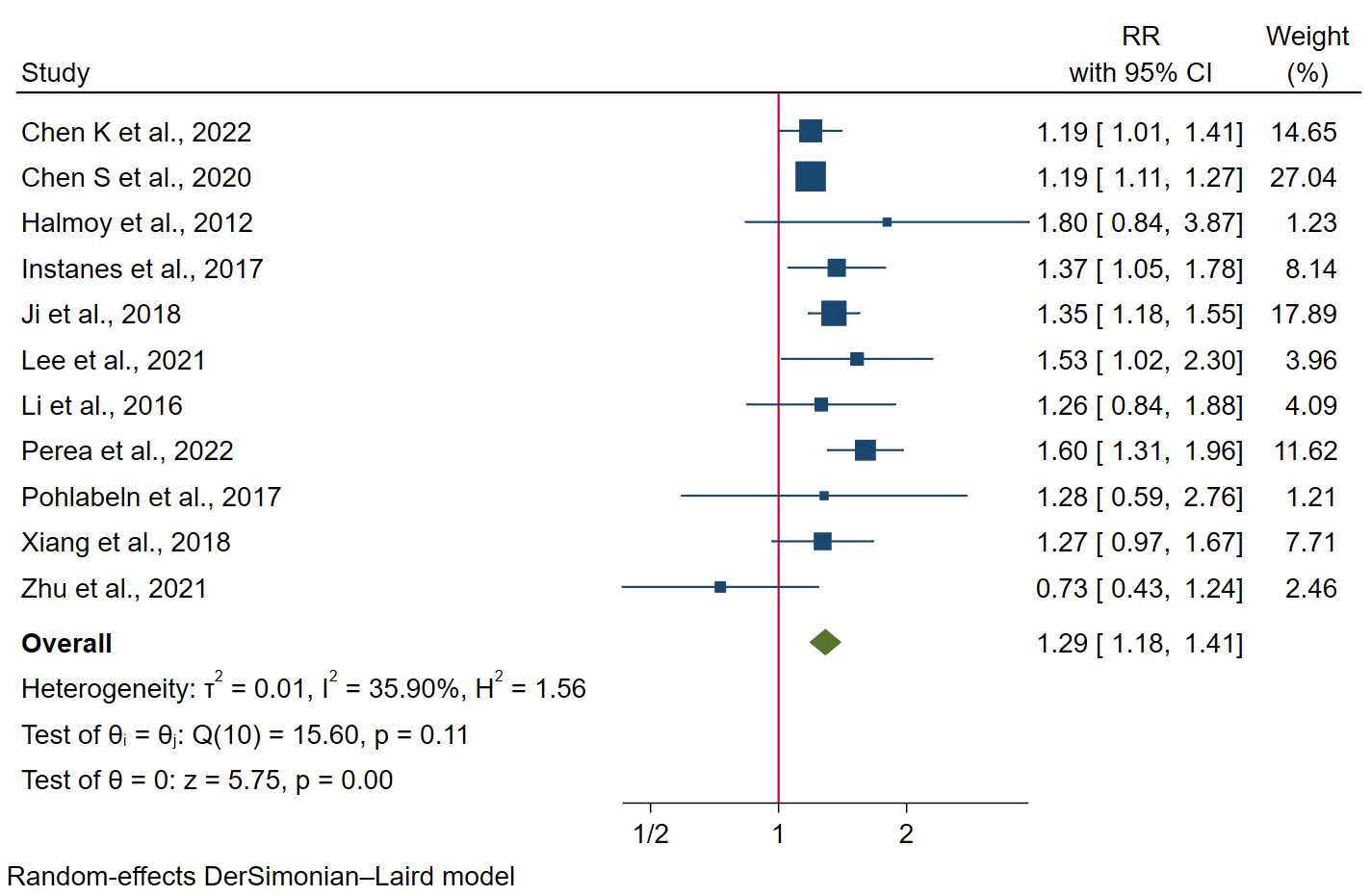


**Figure S3: S**ensitivity analysis removing six studies with small sample size (n<1000).


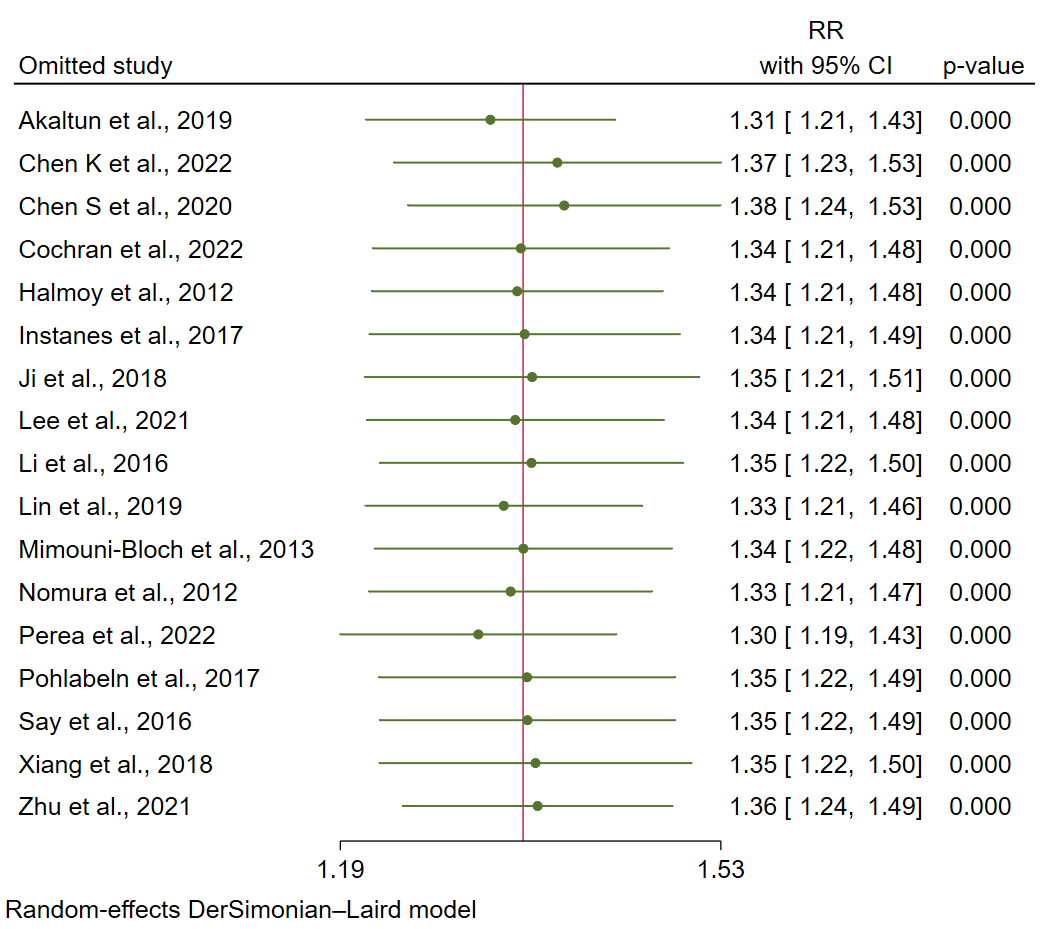


**Figure S4:** leave-one-out sensitivity analysis to assess single study effect on the association between maternal diabetes and the risk of ADHD in offspring.


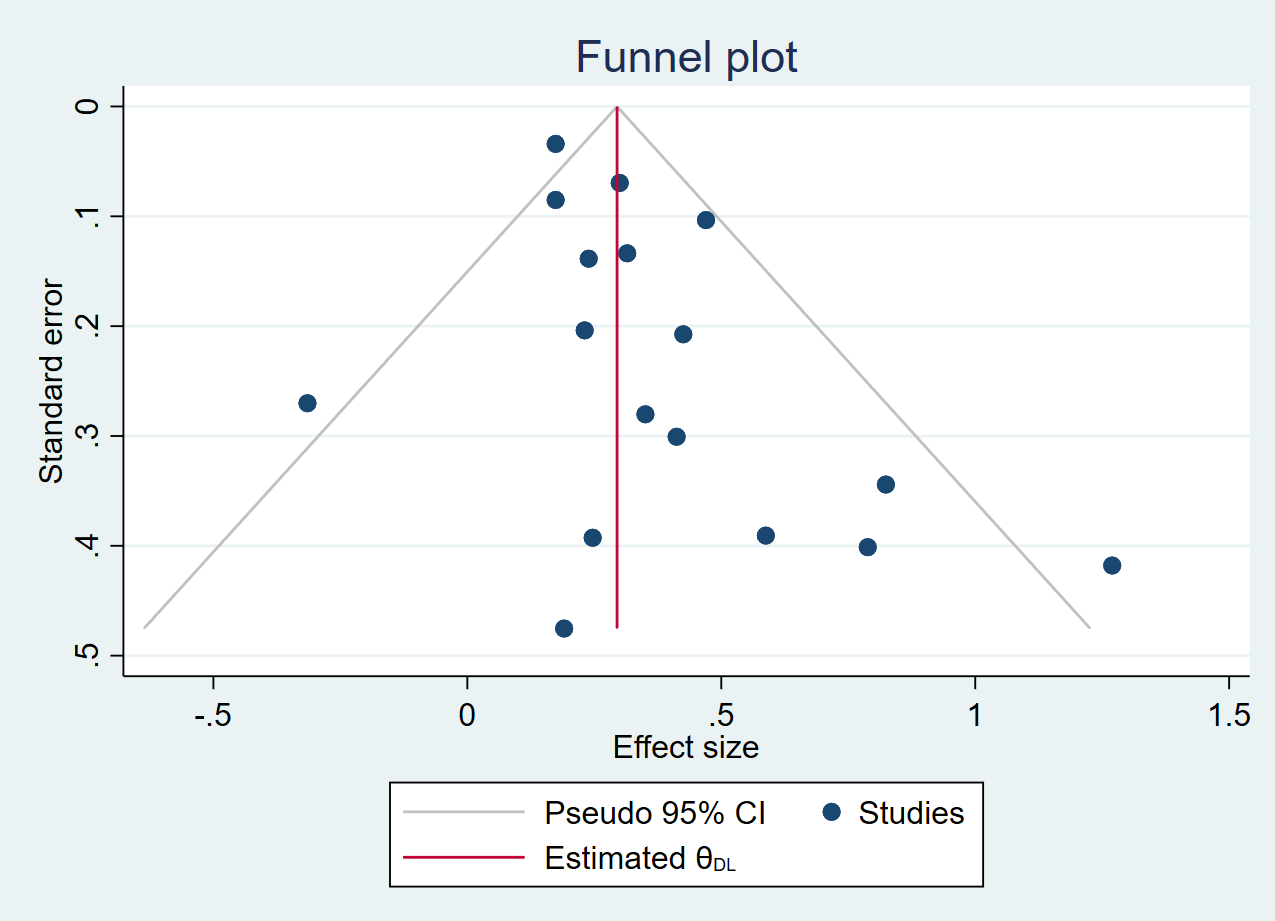


**Figure S5:** Funnel plot showing publication bias among studies examining any form of maternal diabetes and the risk of ADHD in offspring.


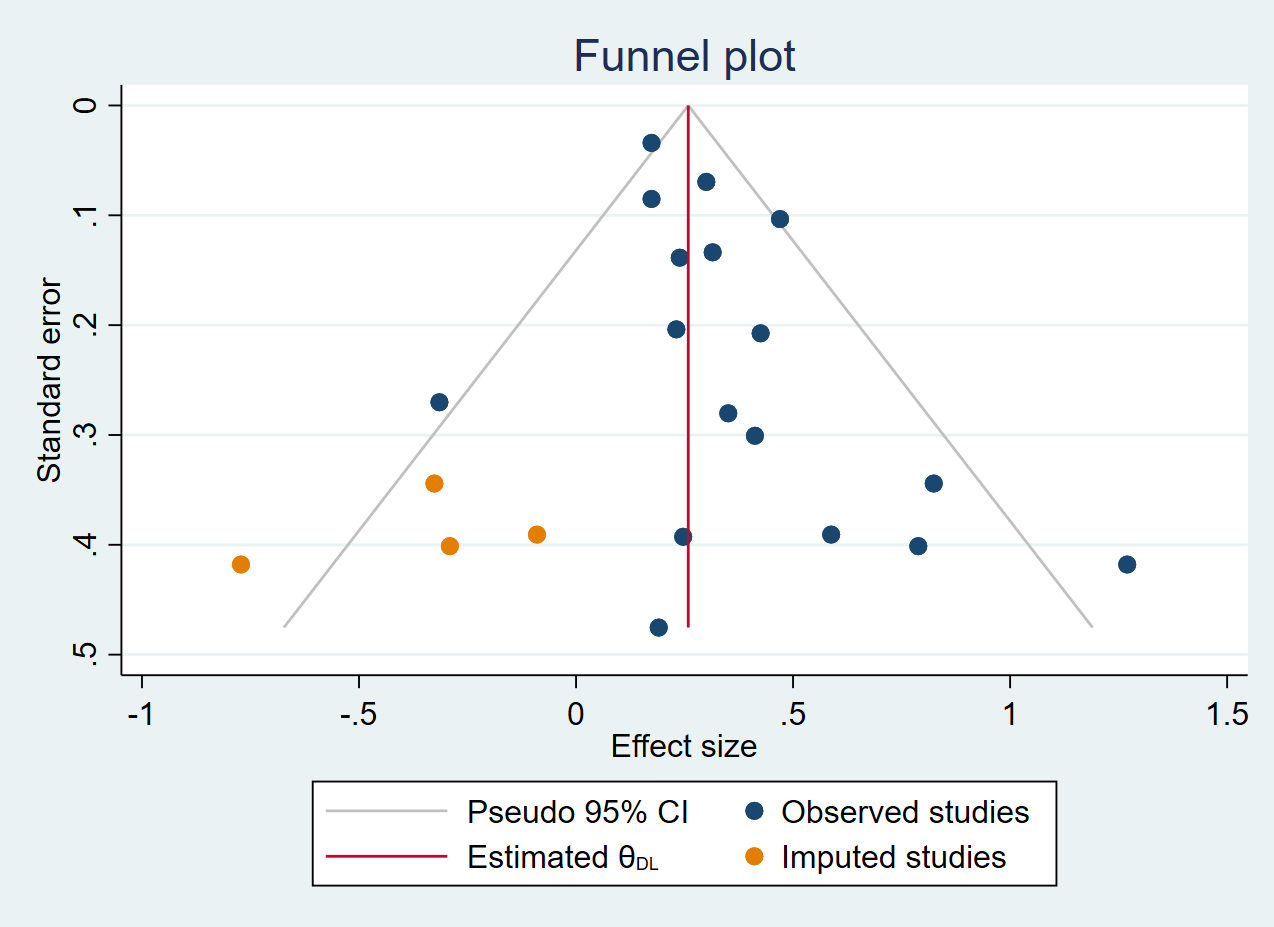


**Figure S6:** Funnel plot showing studies imputed for trim-fill analysis.
